# Is single-stage bilateral medial opening wedge high tibial osteotomy advisable?

**DOI:** 10.1186/s12891-024-07501-2

**Published:** 2024-06-26

**Authors:** Peizhi Yu wen, Huilian Sun, Jiaqi Li, Chunxu Fu, Pengzhao Chen, Jiahao Yu, Wei Chen, Yingze Zhang

**Affiliations:** 1 Orthopaedic Research Institute, Shijiazhuang, Hebei Province People’s Republic of China; 2 Trauma Emergency Center, Shijiazhuang, People’s Republic of China; 3 Department of Orthopedic Surgery, NHC Key Laboratory of Intelligent Orthopaedic Equipment, The Third Hospital of Hebei Medical University, No. 139 Ziqiang Road, Qiaoxi District, Shijiazhuang, 050051, People’s Republic of China; 4 Hebei Orthopaedic Clinical Research Center, Shijiazhuang, People’s Republic of China; 5 Engineering Research Center of Orthaepedic, The Third Hospital of Hebei Medical University, 139 Ziqiang Road, Shijiazhuang, 050051 People’s Republic of China; 6 The Second Hospital of Hebei Medical University, No. 215 Heping West Road, Shijiazhuang, 050004 People’s Republic of China

**Keywords:** Knee osteoarthritis, Opening-wedge proximal tibia osteotomy, Safety

## Abstract

**Purpose:**

To validate the safety and clinical results of single-stage bilateral versus unilateral medial opening wedge high tibial osteotomy (HTO).

**Methods:**

A propensity-matched cohort study was performed from March 2020 to March 2021 in our medical center. Data were prospectively collected. Including 34 patients who underwent single-stage bilateral medial opening HTO(SSBHTO), and 68 cases in the unilateral group. Propensity-matched ration was 2:1 based on age, sex, and body mass index using R software. Comparisons of the length of hospital stay, operative time, blood loss, postoperative adverse events, 90-day readmission rate, conversion to TKA rate, self-reported VAS and WOMAC scores were made to investigate the safety and clinical results of bilateral HTO.

**Results:**

The mean length of hospital stay was 7.36 ± 2.23 days for SSBHTO and 7 days (IQR, 3 days; range, 4 to 23 days) for the unilateral group (*P* = 0.219). The mean operative time was 144 ± 47 min for bilateral HTO and 105(37.5) mins for a unilateral OWHTO (*P* < 0.001). The mean blood loss was 150(100) ml for SSBHTO and 100(50) ml for unilateral OWHTO (*P* < 0.001). There were no significant difference of the adverse events and 90-day readmission rate between two groups. No failed HTO or conversion to knee arthroplasty were observed at the end of follow-up. VAS, pain, stiffness, and functional scores of the WOMAC scale were essentially comparable of two groups one year after surgery (*P* > 0.05).

**Conclusions:**

A single-stage bilateral medial opening wedge high tibial osteotomy is advisable for patients with knee osteoarthritis. Patients benefit from avoiding secondary anesthesia, postoperative complications, and substantial cost savings.

**Level of Evidence:**

Therapeutic Level III.

Osteoarthritis (OA) directly leads to joint pain and is often accompanied by functional deficits, reduction of life quality, and consequently reduced patients’ life expectancy [3, 10, 13]. Knee osteoarthritis (KOA) is by far the most common site of OA lesions, affecting not only the senior population, but all age groups [17], and invades their physical activities at different levels [22]. Medial Opening Wedge High Tibial Osteotomy (OWHTO) is a standard operative option for KOA. It offers a solution for patients who are too afraid of knee arthroplasty and for those with less severe conditions. Interestingly, most varus alignment does not occur in isolation, so alignment correction on both limbs in KOA patients may be ideal [9, 18], but 1-stage surgery by far is the most popular choice. Several studies have focused on this clinical question and reported a shorter hospital length of stay (LOS) and less risk of complications of simultaneous bilateral HTO (SSBHTO) [4, 9, 18]. However, patients’ acceptance and safety concerns remain in regard to the increased risks of perioperative complications such as venous thromboembolic events (VTEs) and blood transfusions [4, 11]. In our medical center, many patients do not have confidence in SSBHTO and prefer unilateral HTO, and physicians are reluctant to take the risk. Therefore, we conducted this study to verify the safety and clinical results of simultaneous and unilateral HTO.

## Materials and methods

A total of 1,312 OWHTO cases were included in our study from March 2020 to March 2021, only 34 patients (2.6%) accepted SSBHTO. The SSBHTO group was then matched to the unilateral HTO in a 1:2 ratio based on age, sex, and body mass index using R software. Inclusion criteria were primary isolated medial compartment OA, with no age restriction. Exclusion criteria were active infection of lower limb, grade 4 of K&L scale, fractures around the knee or concomitant ligament reconstruction, femoral-tibial angle (FTA) > 185° and flexion contracture > 15°, and extreme obesity (BMI > 40 kg/m^2^). K-L classification, Anesthesiologists (ASA) classification, hospital length of stay (LOS), blood loss, operative time, rate of adverse events (AEs), 90-day readmission rate, conversion to TKA rate, preoperative and 3-month postoperative hip knee ankle angle(HKA), preoperative and 1-year postoperative visual analogue scale(VAS) and The Western Ontario and McMaster Universities (WOMAC) score were compared.

All experiments were performed in accordance with relevant guidelines and regulations. The study was approved by the Ethics Committee of the Third Hospital of Hebei Medical University (2021–056-1). Informed consent was obtained from all patients and/or their legal guardian(s).

**Fig. 1:**
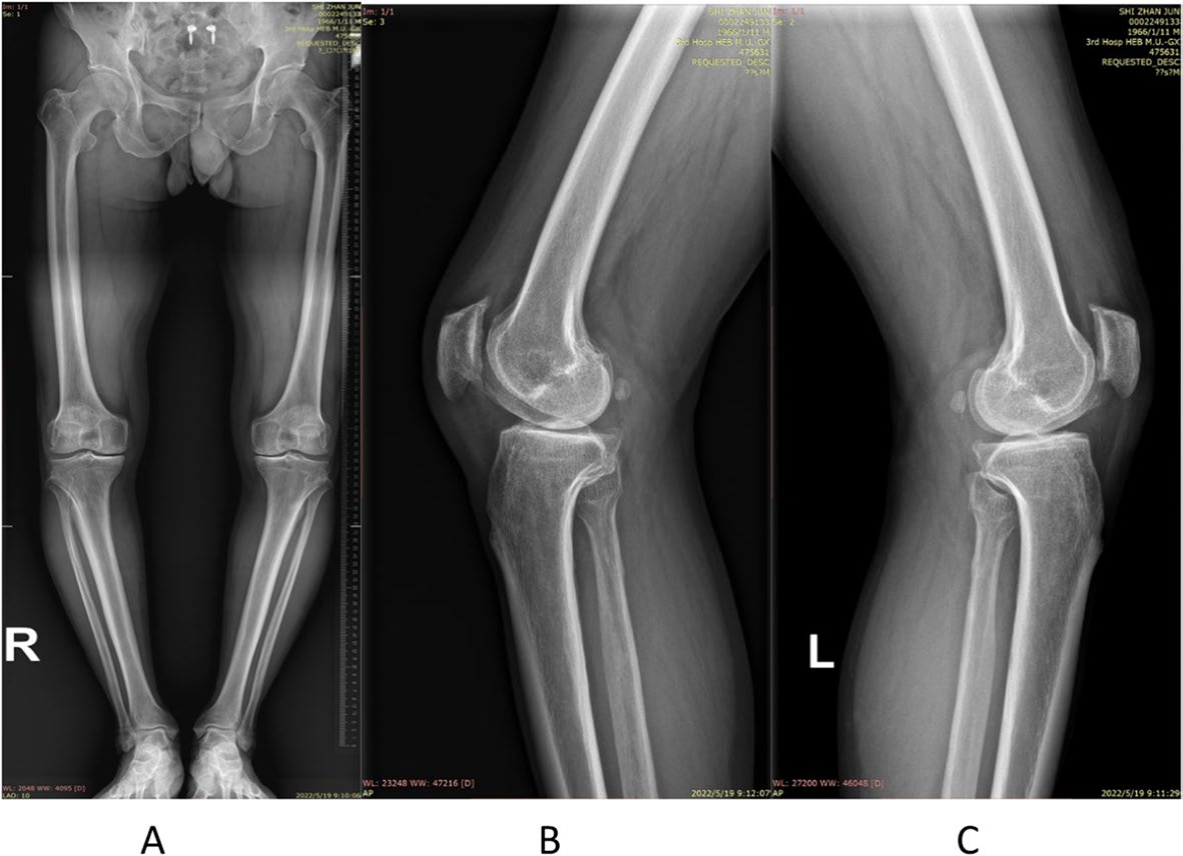
**A**-**C** Weight-bearing full-length radiographs of the lower limb and knee lateral x-ray before bilateral THO

**Fig. 2:**
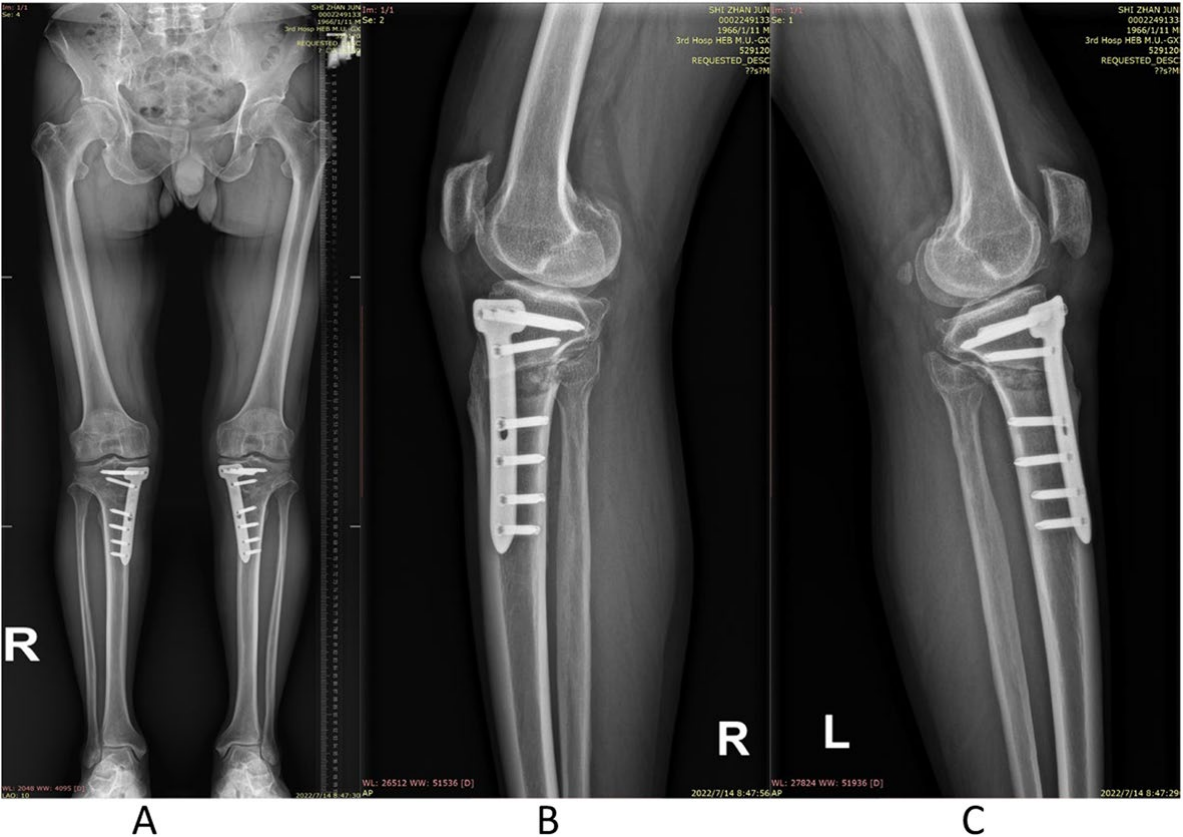
**A**-**C** Weight-bearing full-length and lateral radiographs of the lower limb and knee lateral x-ray after bilateral THO

The preoperative planning and surgical procedures were performed as previously described [2, 5, 6, 14–16]. A 6–8 cm skin incision is made in the anterior medial 1/3 of the tibia from the insertion of the pesanserinus to the posteromedial corner of the tibial head. Care was taken to preserve the infrapatellar branch of the saphenous nerve. The deep subcutaneous fascial agent is partially separated, and the posterior medial cortex of the proximal tibia is completely exposed. Two parallel k wires are drilled towards the apical of the fibula to mark the direction of the osteotomy. After satisfactory intraoperative fluoroscopy, the osteotomy is performed using an oscillating saw along the down edge of guide wires. Once reaching the planned horizontal depth, an anterior ascending cut was performed above the tibial tuberosity using a narrow oscillating saw. The osteotomy expansion devices were inserted, and the gap is gradually and carefully opened. The leg axis is re-evaluated before a long limited contact-dynamic compress plate (LC-DCP, China Vigor) is anteromedially placed under the pesanserinus and the periost [8] (Figs. 1 and 2). Finally all components were tightened and the insicion was closed.

General anesthesia via laryngeal mask, airway and nerve block were the favored techniques. Standard antibiotics (Ancef [cefazolin]) were used at the beginning of operation. Enoxaparin sodium injection was routinely used postoperatively to prevent venous thrombosis of lower limbs.

After completion of the radiological examination, full weight bearing on the affected leg was allowed at 6 weeks postoperatively, and 2 weeks should be extended for bilateral HTO patients [4, 6].

Statistical analyses were performed using SPSS (22.0, USA). Propensity score (PS) were matched using R software (4.1.2). Continuous variables were tested for normal distribution using Kolmogorov–Smirnov test, and data conformed were expressed as ${\overline x}_{\pm s}$, and calculated by an independent sample *t*-test. Otherwise Mann–Whitney U tests was used between groups. Where appropriate, chi-square and Fisher’s exact tests were used to compare categorical variables between groups. *P* < 0.05 were considered significant.

## Results

Thirty-four patients in the SSBHTO group were matched to 68 patients who underwent unilateral HTO. The male/female ratio in the bilateral group was 6/28, average aged was 55.96 ± 5.40 years, and average BMI was 27.24 + 2.84 kg/m^2^. The male/female ratio in the bilateral group was 13/55, average aged was 55.64 ± 5.79 years, and average BMI 26.38 + 2.82 kg/m^2^_._ There was no difference in ASA class, comorbidity, preoperative and 3-month postoperative HKA, preoperative VAS or WOMAC scores between groups (*P* > 0.05). The demographics of two groups are shown in Tables 1 and 3.

**Table 1:** Demographics of propensity-score matched patients

	Bilateral group (*N* = 34)	Unilateral group (*N* = 68)	*P* value
Sex(M/F)	6/28	13/55	0.857
Age (yr)	55.96 ± 5.40	55.64 ± 5.79	0.920
BMI (kg/m^2^)	27.24 + 2.84	26.38 + 2.82	0.110
ASA 1 and 2	32	66	0.471
ASA 3 and 4	2	2	
K-L classification			0.933
1 Grade	2	5	
2 Grade	20	41	
3 Grade	12	22	
Comorbidity (no.[%])			0.966
High blood pressure	12(35.3%)	19(27.9%)	
Diabetes	3(8.8%)	7(10.3%)	
Cardiovascular disease	3(8.8%)	5(7.4%)	
High cholesterol	4(11.8%)	10(14.7%)	
Other comorbidity^a^	28(82.4%)	53(77.9%)	

^a^Kidney disease, liver disease, bone diseases, or “other” disorder

The average LOS was 7.36 ± 2.23 days for the SSBHTO group and 7 days (IQR, 3 days; range, 4 to 23 days) for the unilateral group (*P* = 0.219). The mean operative time was 144 ± 47 min for SSBHTO and 105 (37.5) mins for a unilateral OWHTO (*P* < 0.001). The mean blood loss was 150 (100) ml for SSBHTO and 100(50) ml for a unilateral OWHTO (*P* < 0.001).

Union of the osteotomy gap was achieved in all patients at the time of the final follow-up. None had a second knee arthroplasty. There was no significant difference in deep vein thrombosis (DVT), surgical site infection (SSI), hinge fracture, anemia, hypoalbuminemia, hypokalemia, hyponatremia, or the 90-day readmission rate between SSBHTO and unilateral HTO. No blood transfusions were given in either group (Table 2). VAS, pain, stiffness, and function scores of the WOMAC scale at 1 year post-operatively showed no significant differences (*P* > 0.05). ( Table 3).

**Table 2:** Comparison of characteristics of SSBHTO and unilateral HTO

	Bilateral group (*N* = 34)	Unilateral group (*N* = 68)	*P* value
Blood Loss (Ml)	150 (100)	100 (50)	< 0.001^#^
Operation Time (Min)	144 ± 47	105 (37.5)	< 0.001^#^
Length of hospital stay	7.36 ± 2.23	7 (3)	0.219
Deep Vein Thrombosis (No.[%])	8(23.5%)	8 (11.8%)	0.262
Surgical Site Infection(No.[%])	1 (2.9%)	0	0.340
Hinge Fracture (No.[%])	3 (8.8%)	4 (5.9%)	0.687
Anemia (No.[%])	4 (11.8%)	1 (1.5%)	0.053
Hypoalbuminemia (No.[%])	5 (14.7%)	2 (2.9%)	0.095
Hypokalemia (No.[%])	4 (11.8%)	1 (1.5%)	0.053
Hyponatremia (No.[%])	2 (5.9%)	1 (1.5%)	0.270
90-Day Readmission Rate	1 (2.9%)	0	0.340

^#^*P* < 0.05

**Table 3:** Comparison of VAS and WOMAC scores between two groups

	Bilateral group (*N* = 34)	Unilateral group (*N* = 68)	*P* valus
Preoperative			
VAS	7 (1)	7 (2)	0.418
WOMAC			
Pain	14 (3)	15 (3)	0.085
Stiffness	0 (1)	0 (1)	0.186
Function	53.1 ± 6.2	51.6 ± 6.2	0.239
Total score	67.7 ± 6.8	66.8 ± 6.7	0.642
1 year post-operative			
VAS	2 (3)	1 (3)	0.710
WOMAC			
Pain	2 (3.25)	3 (6)	0.537
Stiffness	0 (0)	0 (1)	0.168
Function	27.9 ± 6.2	25.8 ± 4.8	0.129
Total score	30.4 ± 6.4	29.4 ± 6.4	0.375
HKA			
Preoperative	169.3 + 2.4	168.7 + 2.7	0.821
3 month after operation	177.3 + 1.7	177.4 + 1.8	0.790

*VAS* Visual analogue scale, *WOMAC* Western Ontario and McMaster Universities, *HKA* Hip knee ankle angle

## Discussion

Medial opening wedge high tibial osteotomy (OWHTO), with its ability to preserve complete knee function, has received increasing worldwide attention and is now one of the standard procedures in the treatment of KOA [2, 4, 6, 8, 11, 15, 18]. It corrects coronal malalignment by propping a wedge of bone medially from the proximal tibia, thereby altering the alignment of the knee in an attempt to redistribute the load on tibiofemoral joint [2, 4, 5, 11, 14]. Recommendations for this procedure are derived from a careful evaluation of subjective symptoms, physical examination, and radiographic evidence of arthritis and varus deformity of the lower limbs [8].

It is well known that KOA often affects both knees, a feature that may be particularly true for individuals with substantial bilateral varus alignment [11]. Therefore, SSBHTO is not new, but the disadvantage of this simultaneous procedure is the longer rehabilitation time, with full weight-bearing lasting about 6 weeks or more after surgery [15]. Thus, 2-staged HTO was the only realistic option for patients with bilateral OA knee for a long time. However, with the advent of reliable implants, patients were able to undergo an early active rehabilitation program and achieve a full weight-bearing walk in 3 weeks after surgery [1, 7, 12, 19–21]. Consequently, performing SSBHTO could be beneficial for KOA patients, requiring only one hospitalization, suffering postoperative complications once, and having substantial cost savings. So we performed this propensity-matched cohort study and found that SSBHTO is comparable to unilateral surgery regarding the safety and clinical outcomes.

The average LOS of two groups is around 8 days, unlike Ogawa who reported a 1-week longer hospital stay for SSBHTO than for staged bilateral OWHTO [15]. The operation time of SSBHTO is 144 min, longer than the 105 min of unilateral group, but not double, time saving is mainly in anesthesia and posture placement, and there ia a larger blood loss for SSBHTO (150 vs. 100 ml). Our resluts agreed with Hernigou’s [4] report, that the staged bilateral HTO had greater blood loss and a 35% prolongation of both anesthesia time and time in the operating room.

The main AEs in our cohort were DVT, SSI, hinge fracture, anemia, hypoalbuminemia, hypokalemia, and hyponatremia. The highest incidence was the postoperative DVT (23.5%) in SSBHTO group, and even though no statistical difference were observed of AEs, the incidence of DVT, anemia, hypoalbuminemia and hypokalemia were more than twice as high in the SSBHTO group than in the unilateral group. We believed that SSBHTO is a significantly more traumatic procedure and patients present distinct postoperative frailty. Therefore, surgeons should be kept informed of the general postoperative status of patients as well as their nutrition, not only to the orthopedic-related complications. Hernigou had also revealed a higher risk of transfusion in patients who underwent SSBHTO [4], while no blood transfusion was given in our study due to the mild anemia.

We conducted the VAS and WOMAC scale surveys preoperatively and 1 year after surgery, and found no differences in pain, stiffness and function between the two groups. Hiroyasu Ogawa [15] published a desired functional improvement of both SSBHTO and unilateral HTO at approximately 1 year, but unilateral HTO did not significantly improve until the completion of the second-stage surgery. Our study confirmed the functional improvement of patients at 1 year, but could not explain the long-term effects. In clinics, we saw a significant proportion of patients with unilateral HTO who returned to seek a second osteotomy after 1–2 years of the previous one, but also some patients with low expectations chose to postpone the second-stage procedure.

This study has the following limitations, 1. A relatively small number of each group; 2. Possible inadequate collection of AEs; 3. Short and discontinuous follow-up. 4. We selected patients with unilateral HTO rather than 2-staged HTO, and the choice of a variable time frame seems better, but our findings give more robust comparative results regarding safety and efficacy. We subsequently considered introducing a better adverse event reporting system and conducting a prospective multicentre, large-scale study to confirm the safety and clinical effectiveness of SSHTO.

## Data Availability

All data generated or analyzed during this study are included in this published article.
